# The Aged Care Onsite Pharmacist (ACOP) program in Australia: a qualitative study to examine key considerations for successful implementation in residential aged care homes

**DOI:** 10.1007/s11096-025-01991-3

**Published:** 2025-08-26

**Authors:** Sara Javanparast, Daria S. Gutteridge, Peter D. Hibbert, Elizabeth Manias, Andrew C. Stafford, Gregory M. Peterson, Gillian E. Caughey, Janet K. Sluggett

**Affiliations:** 1 UniSA Allied Health and Human Performance Unit, C/O Level 8, South Australian Health and Medical Research Institute (SAHMRI), University of South Australia, North Terrace, Adelaide, SA 5000 Australia; 2 Australian Institute of Health Innovation, Macquarie University, Macquarie Park, NSW Australia; 3 Monash Nursing and Midwifery, Monash University, Clayton, VIC Australia; 4 Curtin Medical School and enAble Institute, Faculty of Health Sciences, Curtin University, Perth, WA Australia; 5 School of Pharmacy and Pharmacology, University of Tasmania, Hobart, TAS Australia; 6 Registry of Senior Australians Research Centre (ROSA), South Australian Health and Medical Research Institute (SAHMRI), Adelaide, SA Australia; 7 Caring Futures Institute (CFI), College of Nursing and Health Sciences, Flinders University, Bedford Park, SA Australia

**Keywords:** Implementation science, Long-term care, Medication therapy management, Nursing homes, Older people, Pharmacists

## Abstract

**Introduction:**

The Aged Care Onsite Pharmacist (ACOP) program was recently launched in Australia to enable pharmacists to deliver clinical governance, clinical pharmacy and education services on the ground in residential aged care homes (RACHs). As the program is now being scaled up nationally, it is crucial to understand the complex interactions between various factors at the individual and organisational levels to ensure the program is successfully implemented and achieves its ultimate goal of improving the quality use of medicines in RACHs.

**Aim:**

This qualitative study aimed to explore stakeholders’ perspectives on medication management, perceived value of onsite pharmacists, and key considerations for successful program implementation in RACHs.

**Method:**

We employed a qualitative approach and conducted semi-structured interviews (n = 61) with residents/families, pharmacists, medical practitioners, RACH staff, and individuals involved in policy and planning. Participants with experience working in both metropolitan and rural areas were included. The interviews were audio-recorded, transcribed, and thematically analysed, both inductively and deductively. The Consolidated Framework for Implementation Research informed the design of the study, developing interview schedules and data analysis.

**Results:**

Factors influencing the program implementation were grouped into five themes: (1) Individuals: factors concerning individuals involved in the program; (2) Innovation: factors related to the program design; (3) Process: implementation process actions; (4) Inner setting: factors relating to the organisational context; and (5) Outer setting: factors pertaining to the policy context. Most participants valued the potential contribution of onsite pharmacists. Program flexibility was noted as essential to increase its acceptability, uptake and adoptability. A desire for implementation strategies was evident. Workforce, organisational leadership, infrastructure and resources, and broader policy support were noted as critical for the program’s success.

**Conclusion:**

The ACOP program represents a promising strategy to enhance medication management in RACHs. However, implementation on a large scale necessitates a thoughtful consideration of various interconnected factors at the individual, organisation and policy levels that may affect its uptake, adoptability, and long-term sustainability. This has implications for policymakers and providers at the scale up phase to ensure the program achieves its ultimate goal of enhancing residents’ health outcomes.

**Supplementary Information:**

The online version contains supplementary material available at 10.1007/s11096-025-01991-3.

## Impact statements

- The study has identified interconnected factors at individual, organisational, and external levels that should be considered when implementing and evaluating onsite pharmacist services in aged care homes.
- Understanding the perceived enablers and barriers to on-site pharmacist services will help program planners and aged care providers increase program participation and achieve desired outcomes for older adults in aged care homes.
- Other countries can consider these findings when aiming to implement pharmacy practice innovations in aged care homes.

## Introduction

With the population ageing worldwide, the number of individuals living and dying in residential aged care homes (RACHs) is increasing [1]. RACHs (also termed long-term care facilities and nursing homes) provide support for personal and aged care services (either permanently or on a respite basis) to older adults who can no longer live independently [2]. In Australia, between 2017 and 2024, the proportion of people permanently residing in RACHs rose by 6.3% (from 179,000 to 190,000) [3].

Older adults living in RACHs are more likely to experience multimorbidity and have complex medication regimens compared to those living in the community [4]. This results in an increased risk of medication-related errors and harm [5]. Over 95% of residents living in Australian RACHs have at least one medication-related problem [6], which calls for a greater focus on the quality use of medicines in RACHs [7].

Models of pharmacist services have been implemented and evaluated in many countries to improve medication management in RACHs [8], including the Medicine Optimisation in Care Homes program in England [9], and the United States (US) models of Medication Therapy Management [10]. These models have been described in detail elsewhere [8] and have yielded positive outcomes, including improved prescribing, person-centred care, and interprofessional collaboration [11]. In Australia, policy and practice initiatives to support quality use of medicines (i.e., safe, effective, and appropriate use of medicines to achieve the best possible health outcomes [12]) in RACHs include guiding principles for medication management [13], Medication Advisory Committees (MACs) [14], Quality Use of Medicines (QUM) services [12], and government-subsidised Residential Medication Management Reviews (RMMRs) [15]. Furthermore, the recommendations of the recent Royal Commission into Aged Care Quality and Safety in Australia [7], along with the new Aged Care Quality Standards [16], emphasise the need to improve the quality use of medicines and ensure access to allied health services (including pharmacists) in RACHs [7].

### Aged Care Onsite Pharmacist (ACOP) program

In response to the Royal Commission’s recommendations, the Australian Government has made its largest pharmacy-related investment in RACHs (AUD 345 million over 4 years) in the ACOP program, which commenced in July 2024. The program enables a credentialed onsite pharmacist to undertake comprehensive medication reviews, participate in clinical governance, provide training, and assist in monitoring medication usage [17]. RACHs who participate in the program can work with a community pharmacy to provide them with a credentialed pharmacist (Tier 1) or opt to receive a government subsidy to employ a pharmacist directly (Tier 2). The program funds a ratio of one full-time pharmacist per 250 beds [18]. RACHs choosing not to participate can still access services through the existing RMMR and QUM programs.

The effectiveness of ACOPs has been examined in a pilot-controlled trial, which found reductions in the prevalence of inappropriate medication dosages and time spent administering medications [19]. Another mixed-methods study revealed an increase in interprofessional care and relationships [20], and a reduction in potentially inappropriate medication use [21]. Other studies have examined pharmacists’ preparedness to work in RACHs [22]. Investigation into the extent to which the ACOP interventions were delivered as intended revealed a medium level of fidelity [23]. Identified enablers included the provision of information resources and support meetings, recruitment of experienced pharmacists, and a positive RACH culture. Implementation barriers included the complexity of the intervention, a lack of clarity in role descriptions, and the part-time nature of ACOP roles [23].

It is important to understand the complex interactions between unique RACH characteristics (e.g., size, culture, location), organisational factors, and people and their relationships, to ensure the ACOP program is relevant to its context. Failing to recognise these factors could limit the program’s ability to achieve its objective of improving residents’ health outcomes. Despite a series of consultation activities during the ACOP program design [24], there has been limited assessment of the implementation process and the adoptability, flexibility and acceptability of the program through the lens of implementation science theories and frameworks.

### Aim

This qualitative study aimed to explore stakeholders’ perspectives on medication management in RACHs, the perceived value of embedding ACOPs in RACHs, and factors that may influence the successful implementation of the program at scale.

## Method

### Theoretical framework

The study was guided by the Consolidated Framework for Implementation Research (CFIR). The CFIR, widely used in implementation science, emphasises how complex dynamic contexts, either as facilitators or barriers, influence a program’s outcomes in specific settings [25]. According to the framework, successfully implementing an intervention requires a comprehensive understanding of its characteristics and the implementation processes. Moreover, considering contextual factors in both ‘inner’ and ‘outer’ settings, as well as factors related to those who benefit from the intervention, is crucial for identifying and tackling barriers and strengthening enablers [25].

Our study was also informed by a scoping review examining factors (i.e., organisational context, people, relations, innovation characteristics, implementation process strategies) that influence the implementation of innovations in RACHs [26]; these factors align with those described in the CFIR framework. Due consideration of the principles of implementation research are particularly critical in the RACH setting, due to the complexity of its environment, ongoing workforce issues, and evolving policy and political contexts [27].

### Methodology and study design

We employed a qualitative approach and conducted semi-structured interviews with stakeholders with RACH experience. The consolidated criteria for reporting qualitative research (COREQ) was followed [28]. This study was conducted as part of the broader ‘Establishing the PHarmacists Actioning Rational use of Medicines in Aged Care’ (PHARMA-Care) project, which seeks to develop, validate, and test a national quality monitoring program for credentialed pharmacist services in RACHs [29].

In developing the interview schedules, we undertook a review of Australian national aged care and pharmacy policies [7, 13, 14, 16, 30] to identify priority areas for optimal medication management in RACHs. Interviews addressed clinical governance, current practices (e.g., RMMR/QUM), multidisciplinary collaboration, and person-centred care. Participants were asked about the perceived value of the ACOP program, and factors that may enable or hinder its successful implementation. The use of probing questions enabled participants to clarify their stance and therefore address any possibility of social desirability bias [31].

### Participants, recruitment, and data collection

Key stakeholders involved in medicines management in RACHs were recruited through a purposive sampling approach and included residents/families, staff (site managers, nurses) from six metropolitan RACHs maintained by a not-for-profit aged care provider in South Australia, pharmacists with experience in aged care, hospital or community settings, general practitioners (GPs), medical consultants providing services to RACHs, and individuals involved in aged care and/or pharmacy policy and planning across Australia.

For participants from the South Australian RACHs, we sought assistance from the aged care provider to distribute the study flyer to potential participants. The research team contacted participants to provide further information and organise an interview time. For the remaining participant groups, professional networks, research partners, and social media platforms were utilised to promote the study and identify potential participants. All resident/family, and most staff interviews took place face-to-face at their RACH and the rest were conducted online via Microsoft Teams.

We started with a minimum of 5–10 interviews for each participant group to ensure that a variety of perspectives were gathered. We aimed for a higher number of interviews with those who play a more significant role in pharmacist services, such as pharmacists and aged care staff, as well as care recipients, including residents and their families. The final number of interviews was based on accessibility and interest from participants, study timeframe and reaching data saturation when no new insights were obtained from the interviews [32, 33]. Interviews were conducted between November 2023 and May 2024 by an experienced qualitative researcher (SJ). There were no preexisting relationships between the interviewer and participants.

### Analysis

Interviews lasted 40–60 min, were audio-recorded and transcribed verbatim by a professional transcription company and imported into qualitative data management software (NVivo version 14). SJ analysed data by developing a coding framework, utilising an inductive and deductive thematic analysis approach [34]. Guided by our theoretical framework, we first generated deductive codes, followed by codes generated from the data during the inductive analysis. We collected and analysed interview data concurrently, which enabled us to evaluate data redundancy. Data were gathered from multiple perspectives to enhance the validity and credibility of findings. The coding framework and constructed themes were frequently discussed and refined in internal team meetings. We also sought feedback from the wider project governance committees, who had expertise in aged care, pharmacy, consumer advocacy and qualitative methodologies to confirm the accuracy of our interpretations.

### Ethics approval

Ethics approval was obtained from the University of South Australia Human Research Ethics Committee on 8th of November 2023 (ID 205612).

## Results

Of the 73 invited potential participants, seven did not respond, two declined due to a lack of recent aged care involvement, and three agreed to participate but did not confirm an interview. Sixty-one individual interviews (48 females and 13 males) were conducted with 48 participants from South Australia and 13 from other Australian states/territories (Table 1).

**Table 1:** Characteristics of interview participants (n = 61)

Participant group	Number & gender	Experience and other characteristics
Resident Family member	10 (8 females; 2 males) 2 females	Lived experience of using RACH or a family member of a person living in a RACH Age of residents (57 to 99; average 87 years old) Length of stay at RACH between 4 months and 8 years Residents’ main health issues: blood pressure (5), cancer (1), stroke (1), multiple sclerosis (1), dementia (1), kidney problem (1), other cardiovascular (2) 8 transits from home; 4 transits from hospital
RACH staff (site manager, registered nurse, enrolled nurse*, nurse practitioner)	16 (15 females; 1 male)	Providing nursing care to residents in RACHs including medication administration (and prescribing by nurse practitioners) or site manager Work experience in aged care from 1 year to over 30 years
Pharmacists (hospital, community, aged care)	19 (15 females; 4 males)	Providing pharmacist services as a hospital, community or aged care pharmacist or a combination of these in metropolitan and/or rural areas. Three pharmacists had experience working as ACOPs prior to the program commencement Work experience as a pharmacist between 10 and 25 years)
General medical practitioners	9 (5 females; 4 males)	Experience of providing general medical practitioner services to residents in RACHs, general practice and hospital in both metropolitan and rural areas Work experience as a GP between 6 and over 40 years
Specialist medical consultants	2 (1 female; 1 male)	Providing consultation services (geriatrics, palliative care) to RACHs
People involved in policy and planning	3 (2 females; 1 male)	Involved in medication safety policy and programs at national or state/territory levels in metropolitan and/or rural areas

ACOP: Aged Care Onsite Pharmacist; RACH: residential aged care home

^*^Enrolled nurses are registered nursing professionals who have completed a Diploma of Nursing. They work under the direction and supervision of a Registered Nurse (RN)

The qualitative analysis, informed by CFIR and a review of factors influencing implementation of interventions in RACHs [26], identified five key themes regarding the ACOP program implementation: (1) Individuals: factors concerning the individuals involved in the implementation process or affected by the program; (2) Innovation: factors related to the program’s characteristics and design; (3) Process: the implementation process actions; (4) Inner setting: factors relating to the aged care organisational context; and (5) Outer setting: factors pertaining to the external context. These interconnected factors influence one another; therefore, the views and illustrative quotes presented may fit into multiple themes.

### Individuals: factors relating to people who are involved in or impacted by the program

Most participants, especially nurses, appreciated the potential contribution of an ACOP through promptly identifying and correcting medication errors, saving time for the nursing workforce, and providing staff training.

“It [having a pharmacist onsite] is very beneficial because even though we are clinicians, sometimes we don't know the medications; we are not specialised. Sometimes we don't know how to explain it to residents.” (Registered nurse, ID 21)

However, RACH staff views towards the ACOP program partly depended on their existing relationships with the community or RMMR pharmacists and established trust.

“I don't know what an onsite pharmacist is going to add above what we've got at present. We have a good relationship with our local pharmacy and clinical pharmacist. We have a computerised system, so our medical errors are very low.” (Registered nurse, ID 4)

Moreover, attitudes about the role of GPs and pharmacists in medication management, and their authority in decisions about medicines, influenced how some participants perceived the added value of ACOPs.

“From my perspective, there is not much difference between onsite pharmacists and outside community pharmacists. Ultimately, whatever the doctor wants to do it, he’ll do it.” (Enrolled nurse, ID 9)

Similarly, we heard mixed opinions from residents and families. While some acknowledged the opportunity for ‘*more one-on-one contact*’, and ‘*explaining the medicines that residents take*’ if a pharmacist was present, others regarded GPs as the primary individuals responsible for their medications.

Most GPs agreed the opportunity provided by the ACOP program for improved communication and relationship building, ultimately leading to better medication management and continuity of care.

“Certainly, in terms of polypharmacy, it’s very useful to have a backup from a pharmacist explaining and helping you, maybe with a little bit more time than I have, why some of these things are unnecessary.” (GP, ID 11)

Pharmacists with experience providing RMMRs compared the two models, highlighting the advantages and disadvantages of each, with some suggesting a mixed model.

“As an RMMR pharmacist, it was very difficult to change anything from a system level…you weren't there enough to embed yourself into the culture. I don't think you had the credibility because you’re not actually part of the team and could not give much advice.” (Pharmacist, ID 35)

On the other hand, some pharmacists found it advantageous to be part of a larger organisation that provides RMMR services. This offers convenient access to centrally developed educational resources, assistance with data analysis and interpretation, and connections with other pharmacists.

Despite generally positive perceptions about the program, there was limited awareness of its implementation process and the resources available to RACHs and onsite pharmacists at the time of the interviews. RACH staff and pharmacists, who are the key program players, expressed their limited knowledge about the program. There was a prevalent view that more guidance was needed to effectively support program delivery.

“Actually, it's my first time to hear that we could have a clinical pharmacist here.” (Enrolled nurse, ID 20)

None of the residents or families knew about a visiting pharmacist or the ACOP program, including what it would involve, and how they would benefit from it. Nevertheless, after explaining the ACOP role, most felt that having a pharmacist present in their RACH would be highly valuable.

“That would certainly be a help on the site. You can go to them [pharmacists] for any advice, that would be wonderful. In many cases if I have side effects with tablets, I'll go on the internet and find out. I wouldn't need to have to do that then.” (Resident, ID 5)

Table 2 presents the definition, sub-themes and additional quotes related to stakeholders’ knowledge and attitude.

**Table 2:** Definition, sub-themes and additional quotes to illustrate theme 1

Definition	Sub-theme	Additional illustrative, verbatim quotes
Refers to the beliefs and attitudes of aged care stakeholders in relation to the program. It also refers to people’s knowledge and understanding of the program and their skills and competencies to implement the program	Perceived value of the program	“They [RACHs] rely greatly on the supplying pharmacy. Sometimes being on the phone is not very efficient like getting deliveries. It could delay prompt treatment, that’s why they were going to trial a pharmacist on site to help with the medication management side.” (Pharmacist, ID 28) “Personally, I don't understand why a doctor wouldn't be able to tell me about my mum’s medications in the first instance, so that's a bit of a new thing…” (Family member, ID 1)
	Comparing ACOP with RMMR	“We work for a company, and our head office provides statistical data for the RACH. They have time to write the flyers and the educations. As a stand-alone pharmacist, I can sit down and spend my one day a week writing educational content, but then I won't be doing other things… we also have a WhatsApp group to put clinical questions on there. I can ring people and ask ‘what you think of this’? I think, ideally, it would be nice to have a mix of both models.” (Pharmacist, ID 49)
	Knowledge about the program	“I always ring the community pharmacists about medications. What would be the onsite pharmacist’s role in that? Do I go through them as an intermediary, which would be a bit crazy? I don't know what they would be doing and which one to use.” (GP, ID 24) “I’ve never seen a pharmacist here and I don’t know about the program that you are talking about…if I have a question about my medications, I normally ask the nurse or when the doctor comes in, they will explain.” (Resident, ID 13)

### Innovation: factors relating to the ACOP program characteristics and design

Participants emphasised the need for sufficient flexibility in the program design to encourage uptake by RACHs, including rural sites, and larger aged care providers to utilise ACOPs effectively across sites. Flexible working arrangements for ACOPs was another area commented on, mainly by pharmacists.

Other examples related to the program design included the pharmacist-to-bed ratio, minimum pharmacist working hours, and incentive systems that were perceived to be more suited to RACHs in metropolitan than rural areas. Table 3 presents the definition, sub-themes, and illustrative quotes related to the program design.

**Table 3:** Definition, sub-themes and quotes to illustrate theme 2

Definition	Sub-theme	Illustrative, verbatim quotes
Refers to the characteristics and design of the program, its adoptability, flexibility, and compatibility with the aged care context	Flexibility	“I just like to see flexibility if we want to attract pharmacists to do this work… if there's no flexibility, a lot of experienced people who have been working for a long time, you just don’t want them to… [decline].” (Pharmacist, ID 29)
	Incentive system	“I think rural homes will find it more difficult to entice an onsite pharmacist to attend. Whether there has to be an extra allowance for rural travels, the same as an RMMR loading incentive. So, taking the time to travel there and back compensated.” (Pharmacist, ID 52)
	National roll-out	“With any changes, some people are unhappy with how things are done. I feel that maybe a progressive change and rollout would be better than a sudden change. With any new programs being implemented, it would take quite a long time to reach a stable state.” (Pharmacist, ID 28)

### Process: implementation process actions

Overall, there was a desire for detailed information and practical resources to be provided well before the program commencement to help stakeholders learn about the program, ACOP roles, and implementation steps. Pharmacists with previous experience of working onsite in a RACH had a clearer understanding of the roles, implementation enablers and barriers, and potential outcomes. In contrast, other interviewees were generally unaware of the information and resources available, and ways to adopt  the program at their RACH. There was a desire for additional resources, such as implementation toolkits, clinical guidelines and induction checklists.

“There definitely have to be specific guidelines around what we should and shouldn't do as pharmacists working onsite, I think the homes need to be aware, too, of the program, what our process is and what we offer.” (Pharmacist, ID 52)

GPs were another professional group identified as key program players. Participants commented on the need for stronger engagement with GPs and medical professional bodies to build trust and improve their interest and involvement in the program.

“There hasn't been enough collaboration with medical organisations… at times, they’ve come out saying positive statements, but to really inculcate the role, we need some high-level statements from those organisations.” (Pharmacist, ID 31)

At the time of our interviews, participants had limited knowledge of ACOP training requirements. Suggested implementation actions included pharmacist workforce training, establishing ACOP program champions, mentorship initiatives, and a community of practice to facilitate knowledge exchange and experience sharing.

“I think having a network of people who have done the role or are working with the role…Having that sort of mentorship definitely helps.” (Pharmacist, ID 33)

Table 4 presents the definition, sub-themes and additional quotes related to the implementation strategies.

**Table 4:** Definition, sub-themes and additional quotes to illustrate theme 3

Definition	Sub-theme	Additional illustrative, verbatim quotes
Refers to the nature, extent, and quality of strategies and planned actions to promote the program, and to engage with the aged care sector and other stakeholders in program implementation. It also refers to the mechanisms to facilitate program implementation, including training, champions, and knowledge sharing	Implementation strategies	“The program has been talked about for about two years now, but it somehow couldn't be implemented for various reasons. One of them is the lack of implementation strategies. No one really knows how to start because it’s a big change. I've heard that a majority of the pharmacists are supportive of the program, but nobody knows how.” (Pharmacist, ID 32)
	Stakeholders’ engagement	“Building up that trust is important…it may take some time to build up that rapport and trust. That could be frustrating that you wanted to talk to them [pharmacists], but they're there on different days.” (GP, ID 54)
	Mentorship program and community of practice	“A community of practice, I think that's really important. For the people who are working remotely, it's going to be even more important to connect in and be able to debrief, have those mentors, somebody to bounce off because they are going to be working solo as a pharmacist in what can sometimes be a really challenging job.” (Policymaker, ID 55)

### Inner setting: factors relating to the organisational context

The importance of organisational leadership support for the program success was often highlighted, especially by RACH staff.

“When you look at your management or your head office, they need to be open to the program. You do find some people who think, "This is how it's been done for this long, why should we change?"” (Enrolled nurse, ID 7)

Many participants highlighted the importance of sufficient funding for program implementation. While the program covers the ACOP’s salary, participants felt that RACH needs to provide infrastructure, communicate with staff to raise awareness, and manage other necessary changes to integrate the ACOP, and that these contributions can be overlooked.

“Adequate funding is going to be a critical thing… The nursing homes would not want to be involved if they saw that they had to pad it up with their own funding if the government did it inadequately.” (GP, ID 27)

RACH size, type, and geographical location are further organisational factors that may influence the program uptake and implementation. Providers that maintain multiple RACHs in rural areas, which are typically smaller and far apart, may face further complications in terms of time management and building relationships with both staff and residents across sites. Another organisational factor reported was related to system interoperability, information exchange and access to electronic systems. Several participants also pointed out the physical environment and infrastructure in influencing program implementation.

“The space certainly can be a bit of a challenge. At times you'd be in the treatment room and then you'd have to leave and just sit in the lounge or dining room table...” (pharmacist, ID 37)

Table 5 presents the definition, sub-themes, and additional quotes related to internal organisational factors.

**Table 5:** Definition, subthemes, and additional quotes to illustrate theme 4

Definition	Sub-theme	Additional illustrative, verbatim quotes
Refers to the RACH resources, physical environment, infrastructure, leadership style and organisational culture that influence program implementation	Governance and leadership	“The leadership and the governance play a significant role…if the leadership is keen to have the clinical pharmacist onsite and would like to see the improvement in medication management, they can get their staff on board. They can have more participation and positive outcomes from that. But if the leadership is not keen to have this role in place, to get some feedback or do those changes, then it will be challenging to implement this role into that facility.” (Registered nurse, ID 10)
	Funding and resources	“This is an adequate-sized facility, but if it’s too big, we need to look into how we communicate with the team that this is the new role which needs planning and time. That would be a significant factor in the implementation of this new role.” (Registered nurse, ID 10)
	Infrastructure and systems	“Space can be an issue. More and more facilities do have treatment rooms because the doctors are asking for space. I think you need those logistics like access to the software and access to Wi-Fi. That's part of, a product and outcome of truly being integrated into the system… The really practical things that are important…” (Pharmacist, ID 31)
	Characteristics	“I can see how the program looks on a metropolitan site. If you have an aged care with 50 beds, and then you can see your FTE and how often you're filling for this. But if you have 450 residents in 39 rural sites, what that looks like in FTE for pharmacists, it’s not something that’s really going to make a difference. So much of that will be lost in the logistics of getting these pharmacists to the site.” (Policymaker, ID 58)

### Outer setting: factors pertaining to the external policy and regulatory contexts

The adoptability,  equitable access, and outcomes of the ACOP program also depend on the broader policy and structural support systems within which it operates. Interview participants frequently highlighted the contextual factors that should be considered when implementing the program. Recent reforms in aged care policies arising from the Royal Commission was reported as a potential barrier to timely initiation of the ACOP program, as RACHs may prioritise addressing their accreditation requirements. Others viewed current reforms, such as the new Aged Care Act, as a positive shift towards improving quality of care, particularly in medication management, which could aid program implementation:

“Clinical care is part of the aged care standards, so medication management is one of them, and I think it plays a crucial role in everything.” (Registered nurse, ID 10)

A major concern was related to the broader workforce policies, including nurses, GPs, and pharmacist access and distribution in RACHs. Access to sufficient pharmacist workforce was identified as a significant challenge, particularly in rural areas, and that sufficient funding was necessary to entice more pharmacists to work in RACHs.

Participants also noted that the current funding models could affect the program implementation. For example, the current GP funding model does not support adequate time for communication with pharmacists or their participation in MAC meetings to address medication management issues. Table 6 presents the definition, sub-themes, and additional quotes related to the external policy factors.

**Table 6:** Definition, sub-themes and additional quotes to illustrate theme 5

Definition	Sub-theme	Additional illustrative, verbatim quotes
Refers to the broader policy context including aged care workforce, funding models, regulations and policy reforms that impact the program implementation, and its equitable access and outcomes	Aged care reform	“When I first started, psychotropic medication was a lot more widely used and not monitored. Now, we have to show that we have a consultation and review process. So, all these regulations are good. That didn't happen 30 years ago.” (Registered nurse, ID 15)
	Workforce distribution	“As with the supply of GPs regionally, pharmacists also are quite short supply in some areas and potentially having the ability to spend that time in residential care, even if it is paid time, it’s just potentially physically impossible. Just the competing demands on their time.” (Medical consultant, ID 56)
	Funding models	“We could tackle the issue of how we fund GPs, as prescribers, to attend [MAC meetings]; getting GPs at the table for the joint discussions by offering payment for that to happen… that means real-time prescribing and de-prescribing can occur. It is a really efficient means of actually getting those changes in place quickly, but it is hard to replicate unless there's some dedicated funding.” (Medical consultant, ID 56)

## Discussion

Embedding pharmacists in RACHs is a novel clinical pharmacy initiative. Although pilot studies have demonstrated promising results [19, 20, 35, 36], national program rollouts can face challenges. This qualitative study explored key considerations for successfully implementing the ACOP program in Australia. Findings emphasise the significance of sufficient program flexibility, appropriate support for RACHs during the transition, provision of resources and tools, greater awareness about the program among all stakeholder groups, and consideration of internal organisational and external policy factors for the program to succeed. These findings provide valuable insights for program funders, aged care providers and onsite pharmacists on the range of real-world factors that, unlike in pilot settings, can significantly affect the successful program implementation and outcomes. With an increasing number of older adults moving to RACHs, lessons from this study can also inform the planning and actions of other countries considering the implementation of pharmacist-led innovations in RACHs. The findings can also be relevant to similar pharmacist integration programs in different healthcare settings, such as primary and community health care.

Importantly, this study identified no ‘one-size-fits-all’ approach when it comes to implementing multifaceted interventions, such as the ACOP program, in complex settings such as RACHs [37]. Previous work suggests the degree of flexibility permitted within complex interventions can impact their acceptability, uptake and adoptability  [31]. Flexibility ensures that the program addresses local needs, fosters engagement from those responsible for implementation [38], and enables program sustainability and equity in less-resourced contexts.

Secondly, greater attention to the program promotion and implementation processes is required to enhance acceptability and uptake. Despite activities undertaken by the ACOP program funders to seek stakeholder feedback about funding models, role description, training requirements and reporting [17, 24, 39], participants felt that additional resources, such as clinical guidelines and support for credential pharmacists, are required. In past studies [40, 41], strategies such as assessment of organisational readiness for change [42], audit and feedback [43], educational meetings [44], mentorship and community of practice models [45], and resources targeting stakeholders across different implementation phases, have been successfully utilised [46]. Implementation and evaluation of a community of practice to support onsite pharmacists is currently being studied in Australia, to generate evidence of the strategy’s effectiveness [45].

Our findings align with prior research suggesting that organisational capacity, resources, and key stakeholders’ attitudes and knowledge can support or hinder the implementation of health and aged care innovations [26, 47]. Despite perceived value of the ACOP program voiced by most participants, their understanding of the key guiding steps for initiating an ACOP were generally lacking. Our findings suggest greater promotion of existing tools [48] and additional practical resources for RACHs to help them initiate the implementation process. These can be designed with a focus to identify and address key internal organisational and external factors influencing ACOP program implementation [49]. Plain language resources specifically for residents and families are also important to increase awareness of the credentialed pharmacist services that they can access in RACHs.

Lastly, to maximise the uptake and success of multifaceted and complex programs, it is recommended that, in collaboration with stakeholders, evaluation frameworks, tools, and logic models grounded in implementation science theories are developed [27]. This study provides information about a range of factors that should be considered in developing an evaluation plan and impact metrics for the ACOP program. Evaluation must be incorporated in the initial program design and adequately funded before the program’s launch. Process evaluation, in particular, could assist in continuously improving the program, by identifying and overcoming implementation barriers and, thereby, improving program effectiveness [50].

### Strengths and limitations

Key strengths include the use of CFIR as a common implementation science framework that guided our data collection, analysis, and reporting. This inclusion enabled the exploration of a range of interrelated factors influencing the program implementation and long term sustainability. This addresses a key gap in existing studies examining pharmacist interventions, where few have utilised implementation theories [27]. Another strength is the inclusion of diverse participant groups, particularly residents and families, which enabled the collection of various perspectives. However, residents and RACH staff were recruited from a single not-for-profit aged care provider. To address this limitation, we ensured that other participant groups included individuals with experience across a range of different RACH settings. To ensure trustworthiness, the study design, data collection, analysis, and interpretation were regularly discussed within the project team.

The timeframe of this study presented both opportunities and challenges. Conducting interviews prior to ACOP program commencement was advantageous for examining strategies relating to program promotion, and RACHs and pharmacists support to prepare for this initiative. However, it was impossible to seek participants’ views on any additional information released immediately before program commencement. Some factors discussed represented participants’ perceptions prior to the program implementation.

## Conclusion

The ACOP program represents a promising strategy in enhancing pharmacist services in RACHs. Our findings suggest that there is no one-size-fits-all approach for the program implementation. Flexibility is particularly critical, in addition to implementation guides, early engagement with stakeholders, and considering internal organisational and external policy factors. The program success at a national scale necessitates a thoughtful consideration of these interconnected factors. This consideration is vital for the program to achieve its intended outcome of improving medication management and health outcomes for older adults living in RACHs.

## Supplementary Information

Below is the link to the electronic supplementary material.

Supplementary file1 (DOCX 20 KB)

## Data Availability

Qualitative data generated and/or analysed during the current study are available from the corresponding author upon reasonable request.
