# Supplementary material for: The Aged Care Onsite Pharmacist (ACOP) program in Australia: a qualitative study to examine key considerations for successful implementation in residential aged care homes
Source: Int J Clin Pharm. 2025 Aug 26;48(1):169–81. doi: 10.1007/s11096-025-01991-3 (PMC12823688; doi:10.1007/s11096-025-01991-3)
Supplement: Supplementary file 1 — Supplementary file1 (DOCX 20 KB) [file 11096_2025_1991_MOESM1_ESM.docx]

**Semi-structured interview guide – Pharmacists/doctors**

1. Can you provide me with an overview of your role in medicines management at RACFs?

*Potential prompts:*

- What kind of information exchange do you have with others (e.g., RACF staff, other health professionals, residents?)
- What are some of the problems and challenges you face with the medicines management in RACFs?

1. What aspects of quality use of medicines in RACFs are important to you?

*Potential prompts:*

- How would you describe the RACF medication management culture?
- How do you measure the quality of medicines use in an RACF?
- How does legislation/policy/accreditation facilitate or provide a barrier to quality use of medicines in RACFs?

1. Have you been involved in medication reviews yourself, and if so, how many?

*Potential prompts:*

- Do these services vary across different RACFs?
- How well does this service meet your needs? Are there ways in which you think these services could be improved?
- For pharmacists: How do you determine whether your recommendations have been acted upon and what the outcomes are for the resident?

1. [Explain new onsite pharmacist program]. What pharmacist services are important to you and/or your residents?

*Potential prompts:*

- What model of pharmacist care exists in your RACF? How will you go about implementing an onsite pharmacist model (if you intend to do so)?
- For doctors: How would you communicate and work with the pharmacist in the RACF?

1. What evidence would you consider as being important to show that this new model of pharmacist care is working well?

*Potential prompts:*

- How would you assess the quality of the pharmacist services provided?
- What changes would you expect to see over time in the RACF?

1. Quality indicators are often used to look at the safety and quality of care. What is your experience with using quality indicators in your usual clinical practice?

*Potential prompts:*

- What is the ease of access to indicator results and explanations of the findings?
- Do you (and RACF staff) have the skills to interpret indicator results and implement changes?

1. Is there anything else you would like to discuss that we have not talked about in this interview?

**Semi-structured interview guide –residents of RACFs or family members***

1. Can you tell me about what medicines you take?
2. Do you look after your medicines yourself or do staff help you?

*Potential prompts:*

- - What is working well?
  - What could be improved?

1. How involved would you say are you in the decision-making of the medicines you/ your family member receives?

*Potential prompts:*

- - If there is a change in your medicines, is this discussed with you?
  - If you have any concerns relating to your medicines, such as side effects, who would you talk to about it?

1. Do you know if you have had a medication review from a pharmacist before?

*Potential prompts:*

- - Can you talk to a pharmacist when you need to?

1. How often do you think your medicines should be looked at to make sure everything is working in the best way possible?
2. What would you like to see/change in terms of the management of medicines at your care facility?

*Potential prompts:*

- - Prompts to reflect on the different aspects of medication management (e.g., prescribing, dispensing, administration, education, hospital transfers)

1. [Explain new onsite pharmacist program]. What are your thoughts on this new program? What are the important things for us to consider when we are looking at the impact that this program has for residents such as yourself?
2. Is there anything else you would like to discuss that we have not talked about in this interview?

*Residents and family members may be asked to share their views by telling a story or sharing a specific example that relates to their experiences in relation to the questions above.

**Semi-structured interview guide – RACF staff**

1. Can you provide me with an overview of your role in the medicines management in the RACF?

*Potential prompts*:

- Does your work involve administering medicines? Do residents or their families ever ask you for information about their medicines?
- What are some of the problems and challenges you face with the medicines management in RACFs?

1. What aspects of quality use of medicines in RACFs are important to you?

*Potential prompts:*

- How would you describe the RACF medication management culture?
- How do you measure the quality of medicines use in an RACF?
- How does legislation/policy/accreditation facilitate or provide a barrier to quality use of medicines in RACFs?

1. What pharmacist services are provided in your RACF? Have you been involved in any medication reviews from the pharmacist?

*Potential prompts:*

- Do these services vary across different RACFs?
- How well does this service meet the needs of your organisation? Are there ways in which you think these services could be improved?
- What barriers are there to receiving pharmacist services in your organization?

1. [Explain new onsite pharmacist program]. What pharmacist services are important to you and/or your residents?

*Potential prompts:*

- What model of pharmacist care exists in your RACF? How will you go about implementing an onsite pharmacist model (if you intend to do so)?
- How would you communicate and work with the pharmacist in the RACF?

1. What evidence would you consider as being important to show that this new model of pharmacist care is working well?

*Potential prompts:*

- How would you assess the quality of the pharmacist services provided?
- What changes would you expect to see over time in the RACF because of this program?

1. Quality indicators are often used to look at the safety and quality of care. What is your experience with using quality indicators in your usual clinical practice?

*Potential prompts:*

- What is the ease of access to indicator results and explanations of the findings?
- Do you (and RACF staff) have the skills to interpret indicator results and implement changes?
- What do you do with the results? How much value do you place on the findings?

1. Is there anything else you would like to discuss that we have not talked about in this interview?

**Semi-structured interview guide - Policymakers or people contributing to policy or implementation work**

1. Can you provide me with an overview of your role and how it relates to medicines management in RACFs?
2. What aspects of quality use of medicines in RACFs are important to you?

*Potential prompts:*

- What do you consider important when evaluating the quality of medicines use in an RACF?
- How does legislation/policy/accreditation facilitate or provide a barrier to quality use of medicines in RACFs?

1. [Explain new onsite pharmacist program]. What evidence would you consider as being important to show that this new model of pharmacist care is working well?

*Potential prompts:*

- How would you assess the quality of the pharmacist services provided?
- What changes would you expect to see over time in the RACF?

1. Quality indicators are often used to look at the safety and quality of care. What is your experience with using quality indicators?

*Potential prompts:*

- What do you think the information needs of health professionals and RACF staff might be?
- How do you see quality indicators being used in the future to monitor quality use of medicines in RACFs? What benefits do you hope this could bring and are there any downsides?
- Are there any risks or concerns you have about using quality indicators to monitor the impact of this new pharmacist program?

1. The PHARMA-Care project is focused on developing a set of risk adjusted quality indicators for monitoring quality use of medicines and the new pharmacist program in RACFs.

- How would this initiative fit with wider policy initiatives?

1. Is there anything else you would like to discuss that we have not talked about in this interview?
